# Calculating the potential for within-flight transmission of influenza A (H1N1)

**DOI:** 10.1186/1741-7015-7-81

**Published:** 2009-12-24

**Authors:** Bradley G Wagner, Brian J Coburn, Sally Blower

**Affiliations:** 1 Center for Biomedical Modelling, Semel Institute of Neuroscience and Human Behavior, David Geffen School of Medicine at UCLA, Los Angeles, CA, USA

## Abstract

**Background:**

Clearly air travel, by transporting infectious individuals from one geographic location to another, significantly affects the rate of spread of influenza A (H1N1). However, the possibility of within-flight transmission of H1N1 has not been evaluated; although it is known that smallpox, measles, tuberculosis, SARS and seasonal influenza can be transmitted during commercial flights. Here we present the first quantitative risk assessment to assess the potential for within-flight transmission of H1N1.

**Methods:**

We model airborne transmission of infectious viral particles of H1N1 within a Boeing 747 using methodology from the field of quantitative microbial risk assessment.

**Results:**

The risk of catching H1N1 will essentially be confined to passengers travelling in the same cabin as the source case. Not surprisingly, we find that the longer the flight the greater the number of infections that can be expected. We calculate that H1N1, even during long flights, poses a low to moderate within-flight transmission risk if the source case travels First Class. Specifically, 0-1 infections could occur during a 5 hour flight, 1-3 during an 11 hour flight and 2-5 during a 17 hour flight. However, within-flight transmission could be significant, particularly during long flights, if the source case travels in Economy Class. Specifically, two to five infections could occur during a 5 hour flight, 5-10 during an 11 hour flight and 7-17 during a 17 hour flight. If the aircraft is only partially loaded, under certain conditions more infections could occur in First Class than in Economy Class. During a 17 hour flight, a greater number of infections would occur in First Class than in Economy if the First Class Cabin is fully occupied, but Economy class is less than 30% full.

**Conclusions:**

Our results provide insights into the potential utility of air travel restrictions on controlling influenza pandemics in the winter of 2009/2010. They show travel by one infectious individual, rather than causing a single outbreak of H1N1, could cause several simultaneous outbreaks. These results imply that, during a pandemic, quarantining passengers who travel in Economy on long-haul flights could potentially be an important control strategy. Notably, our results show that quarantining passengers who travel First Class would be unlikely to be an effective control strategy.

## Background

Clearly air travel, by transporting infectious individuals from the epicentre in Mexico to other geographic locations, significantly affected the rate of spread of influenza A (H1N1). However, the possibility of within-flight transmission of H1N1 has not been evaluated, although it is known that smallpox, measles, tuberculosis, SARS and seasonal influenza can be transmitted during commercial flights [1]. These pathogens can be transmitted both by direct contact with large respiratory droplets and by airborne transmission of small, aerosolized droplets [2-4]. Several recent empirical studies have attempted to determine which mode of transmission is the most important for influenza. Fabian *et al*. [2] conducted studies of seasonal strains to measure the concentration of influenza RNA viral particles exhaled by infected patients. In their study, infected patients exhaled into an Exhalair, a device that collects and counts viral particles in exhaled breath, and the concentration of RNA viral copies was measured in Teflon filters. It was found that 33% of infected humans exhaled droplets containing influenza. Lowen *et al*. [3] have experimentally determined that influenza can be transmitted amongst guinea pigs by droplet and/or aerosol spread. In these experiments the guinea pigs were inoculated intranasally and viral samples were collected from nasal washes of their upper and lower respiratory tracts. Viral growth was determined to occur in their lungs and nasal passages, establishing the guinea pig as a model host for droplet infection. Lowen *et al*. [3] also found airborne and droplet transmission could occur between animals by conducting experiments in which they placed infected and non-infected guinea pigs in the same cage, adjacent cages and separated cages at least 90 cm apart [3]. A similar and more recent experimental study using ferrets has shown that H1N1 can be transmitted by aerosolized droplets. Although several studies have shown that H1N1 appears to have similar infectivity as seasonal strains the importance of airborne transmission of H1N1 in humans has not yet been definitively established [4-6]. However, it should be noted that airborne transmission of seasonal strains of influenza during a flight, particularly if ventilation is poor, can be significant. As an extreme example, one individual infected with a strain of influenza A managed to infect 72% of passengers during a 3 hour flight on a plane without ventilation [7]. By assuming that H1N1 has a similar infectivity to that of seasonal strains, we calculated the expected number of H1N1 infections that one source case could cause during an international flight.

Typically, the number of infected individuals that can be expected during a large scale outbreak or epidemic is determined by calculating the basic reproduction number (R_0_) [8]. R_0 _is defined as the average number of secondary infections caused by one infectious individual in a population of susceptible individuals during the time in which they are infectious. Therefore, R_0 _is a useful measure for predicting the number of infections that could be expected to occur during a large-scale outbreak or an epidemic. However, R_0 _is not a useful measure for predicting the number of infections that one infectious individual could cause over a few hours in a confined space. In this case, the number of secondary infections is best predicted using methods from the field of quantitative microbial risk assessment. These are the methods that we have used in our analysis. Specifically, we have used the classic Wells-Riley equation [9] to estimate the number of passengers that could become infected with H1N1 during a commercial flight, assuming one infectious case is on board. The Wells-Riley equation was developed over 30 years ago [9] and is now the standard methodology for predicting (for infectious pathogens that have airborne transmission) the size of outbreaks within buildings and other enclosed environments.

The Wells-Riley equation is based on the number of exposed individuals, the respiratory rate of the source case, the length of exposure to the infectious droplets and the concentration of infectious viral particles over time. Within an aircraft the concentration of infectious viral particles over time is determined by the ventilation rates, the volume of the cabins in the aircraft and the infectiousness of the index case. In the field of quantitative microbial risk assessment, infectiousness is quantified by measuring the number of infectious particles that an infected individual expels per litre of air. Infectivity is expressed in terms of infectious quanta; a measure defined by William Wells in 1934 [10]. Wells used the Poisson distribution to express the relationship between dosage and infection; he defined a quantum to be the number of infectious droplet nuclei required to infect 1 - 1/*e *(that is, 63%) of exposed susceptible individuals. Quanta are now a standard measure for expressing the risk of infection with airborne pathogens and have been used extensively to characterize the infectivity of seasonal stains of influenza [2,11,12]. Rates of quanta production for influenza have been estimated by both direct and indirect means. Indirect infectivity estimates have been made using back calculation based on outbreaks in environments with little or no ventilation [12]. Specifically, Rudnick and Milton [12] used data from an influenza outbreak on a grounded plane with very limited ventilation and a single source case (see Moser *et al*. [7]) to back calculate the rate of quantum generation for a highly infectious case of influenza. Direct measurements of quanta production rates have been made by determining the rate at which infectious individuals exhale RNA virus copies [2]. This is practically accomplished by measuring the RNA virus concentration in Teflon filters in conjunction with an individual's rate of respiration [2].

## Methods

During a flight, transmission of influenza due to direct contact is very limited because passengers remain seated, and therefore we only model airborne transmission. We used a sequential box model [5] to calculate the temporal concentration of infectious droplets (quanta) of H1N1 in a transatlantic airliner with four cabins: First Class, Upper Deck Business Class, Lower Deck Business Class and Economy Class. The sequential box model was developed in the 1980s by Ryan *et al*. [13]. It is a standard method for estimating the concentration of infectious particles in confined environments. The model calculates concentration based on volume of air in each cabin, rate of inflow of air from outside the aircraft, proportion of air recycled during the flight, efficiency of filters in purifying recycled air and strain infectivity. In all our calculations we assume that the index case is symptomatic. If the index case is asymptomatic they would expel very few infectious virions and, hence, would be very unlikely to cause any infections during a flight.

**Figure 1:**
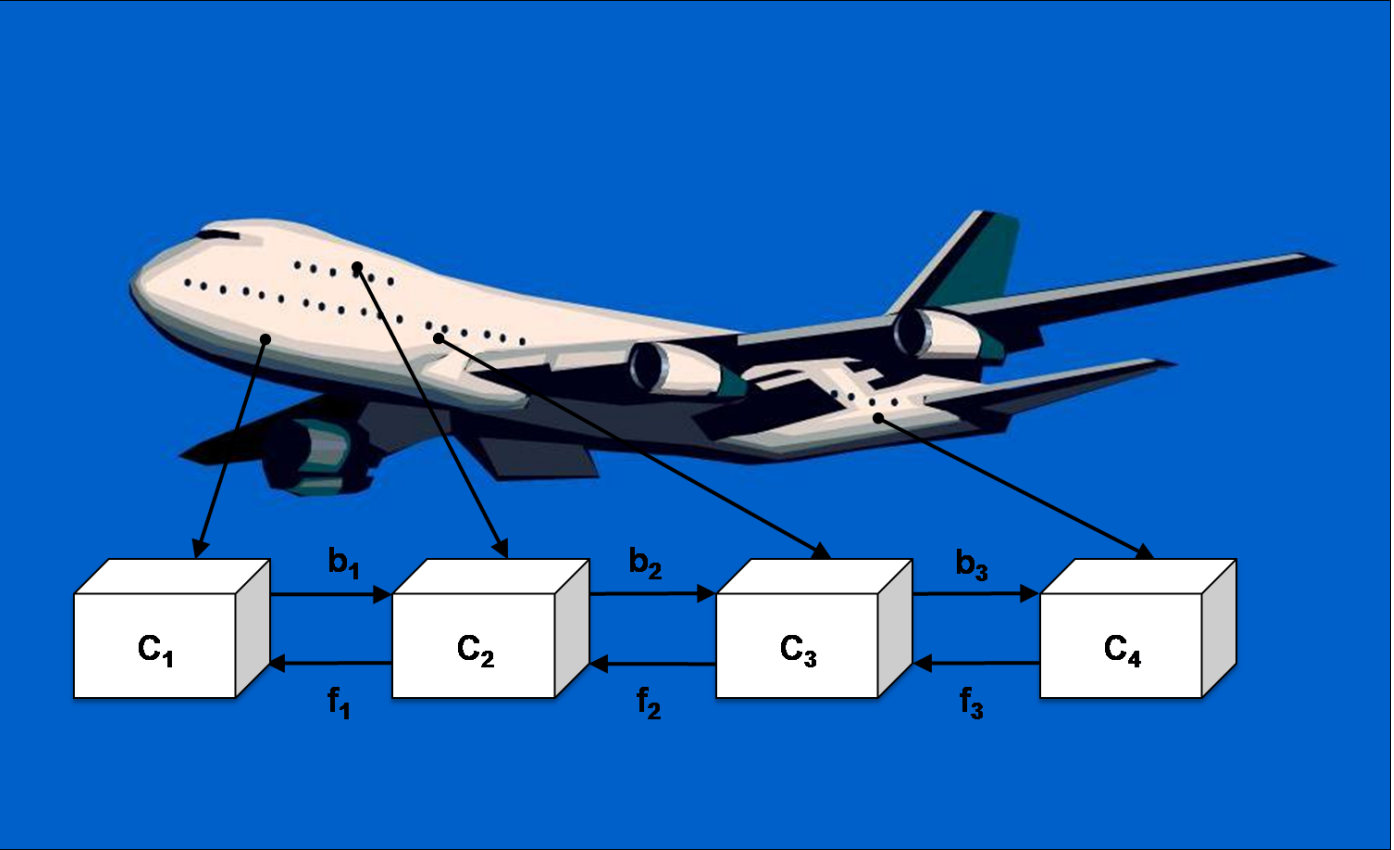
**The Sequential Box Model (SBM) that we used to determine the temporal concentration of infectious droplets of H1N1 in four cabins (C_1_, C_2_, C_3 _and C_4_) in a commercial airliner (specifically, in a Boeing 747)**. Within the aircraft air diffuses between adjacent cabins (forward flow *f*_*i *_and backward flow *b*_*i*_) and is removed and replaced by an air supply system. Air is replaced by outside air and by air that has been re-circulated through a HEPA filter.

Our sequential box model is specified by four equations, one equation for each cabin in the aircraft. The model assumes that the air in each cabin is well mixed. It enables us to track the concentration of infectious quanta of H1N1 (*C*) in each of the four cabins, over time *t*. The subscript to *C *signifies the cabin class: 1 = First Class; 2 = Upper Deck Business Class; 3 = Lower Deck Business Class; and 4 = Economy Class. The sequential box model and the airflow between cabins in the plane are shown diagrammatically in Figure 1. The model is given in terms of a system of ordinary differential equations as:(1)

We model four scenarios, with one infectious individual (index case) in each of the four cabins. The parameter *q*_*i *_represents the rate of production of infectious quanta of H1N1 in cabin i. The subscript to *q *signifies the cabin class. If there is no infectious individual in a cabin *i *then *q*_*i *_is set equal to zero. The infectivity of H1N1 is currently unknown. However, empirical data indicate that it appears to be equally (or slightly less) transmissible than seasonal strains of influenza [5,6]. Therefore, in each scenario we varied the quanta production rate of the index case (*q*_*i*_) from 50 to 128 quanta/hour, based on infectivity estimates for seasonal strains of influenza [2,7,12].

The remaining parameters in the model characterize the environmental conditions in the plane. We parameterized the model to reflect the environmental conditions in a Boeing 747 airliner; parameter values were obtained from Boeing [14]. The rate of forward airflow between adjacent cabins is represented by (*f*_*j *_*j *= 1...3) and the rate of backwards airflow by (*b*_*j *_*j *= 2...4); *f*_*i*_, *b*_*i *_= 61 m^3^/h for all *i*. Air is removed from each of the four cabins at rate *RF*_*j *_*j *= 1...4; *RF*_1 _= 3308 m^3^/h, *RF*_2 _= 2462 m^3^/h, *RF*_3 _= 2284 m^3^/h and *RF*_4 _= 11,679 m^3^/h. A proportion of the removed air (*r*) is filtered with efficacy *α *and then re-circulated: *r *= 0.5, *α *= 0.993. A proportion 1-*r *of the removed air is replaced by air outside the aircraft; the function G(*C*_1_, *C*_2_, *C*_3_, *C*_4_) computes the concentration of infectious quanta in the re-circulated air. The parameter *V*_*j *_represents the volume of air in each of the four cabin classes: *V*_1 _= 133 m^3^, *V*_2 _= 99 m^3^/h, *V*_3 _= 116 m^3^/h and *V*_4 _= 468 m^3^/h.

We then used the Wells-Riley equation [9] to calculate *I*_*i *_(the number of passengers who could be infected in each of *i *cabins during a flight of duration *T*) where the equation is specified as:(3)

where *N*_*i *_represents the number of passengers in cabin *i *and *ρ *represents the volumetric breathing rate of the source case (*ρ *= 0.3 m^3^/h) [15]. We obtained estimates for *C*_*i*_(t), *i *= 1...4 by integrating our sequential box model.  

We set the number of exposed individuals to reflect occupancy in a Boeing 747 operating at full capacity: 23 passengers in First Class; 40 in Upper Deck Business Class; 40 in Lower Deck Business Class; and 313 in Economy. We varied the length of exposure to the H1N1 virus (that is, flight duration) from 5 hours to 11 hours to 17 hours.

## Results

We calculate that the risk of catching H1N1 will, essentially, be confined to passengers travelling in the same cabin as the source case. We found that this result holds across the entire range of infectiousness (q) that we considered. If q is even lower (that is, the source case is even less infectious than we have assumed) transmission to other cabins would be even less likely. Since we have assumed that the air in each cabin is well mixed, individuals in the same cabin will have the same risk of infection.

**Figure 2:**
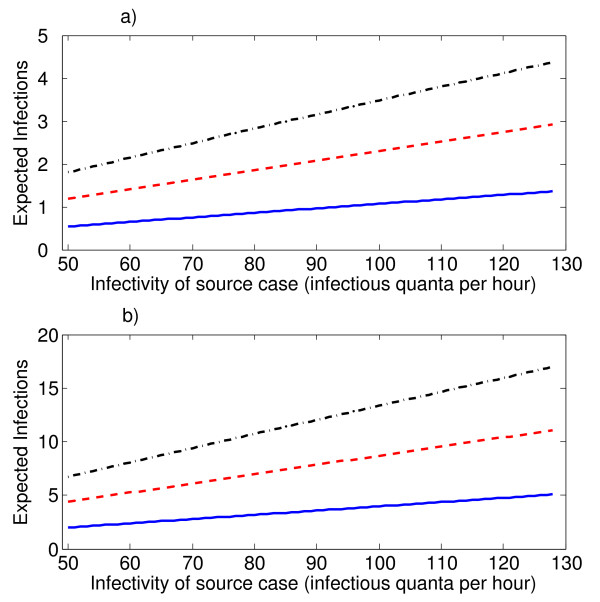
**The expected number of H1N1 infections caused by a single infectious case as a function of flight duration and strain infectivity**. Figure 2a) shows the expected number of infections if the source case is traveling First Class, and Figure 2b) shows the expected number of infections if the source case is traveling in Economy. The three curves in Figures 2a) and 2b) represent different flight durations which are respectively; 5 hrs (solid blue), 11 hrs (dashed red), and 17 hrs (dashed-dot black).

Not surprisingly, we find that the longer the flight the greater the number of infections that may be expected (Figure 2). Our results also show that the greater the infectivity of the source case, the greater the effect of flight duration on increasing the number of infections (Figure 2). This occurs regardless of whether the source case is travelling in Economy or First Class; our results for Business Class are between those for First Class and Economy Class and are not shown.

We calculate that, because of the increased seating capacity of Economy Class, there is a higher probability (75%) that the infectious case will be travelling in Economy rather than in Business (10%) or First Class (5%). Therefore, in general, individuals travelling in Economy are always likely to be at greater risk of infection with H1N1 than individuals travelling in Business or First Class.

Our modelling clearly shows that the location of the source case will have a significant effect on the number of infections that occur during a flight (Figure 2). If the plane is completely occupied, a single infectious individual in Economy Class will cause more infections that a source case in First Class (Figure 2). We calculate that H1N1, even during long flights, poses a low to moderate within-flight transmission risk if the source case travels First Class. Specifically, 0-1 infections could occur during a 5 hour flight, 1-3 during an 11 hour flight and 2-5 during a 17 hour flight (Figure 2). However, our results show that within-flight transmission could be significant, particularly during long flights, if the source case travels in Economy Class. Specifically, 2-5 infections could occur during a 5 hour flight, 5-10 during an 11 hour flight and 7-17 during a 17 hour flight (Figure 2). More infections would occur in the Economy Class than in Business or First Class because the majority of the passengers travel in Economy and that section has the smallest volume of air per passenger. However, if the aircraft is only partially loaded, under certain conditions, more infections could occur in First Class than in Economy Class. For example, we calculate that, during a 17 hour flight, a greater number of infections would occur in First Class than in Economy if the First Class Cabin is fully occupied but Economy class is less than 30% full.

## Conclusions

Our modelling has shown (for the first time) that significant H1N1 transmission could be occurring during air travel. Our results have been obtained from a model parameterized for a Boeing 747. However, our results are generally applicable to other transatlantic aircraft with similar size cabins (in terms of seating capacity and volume) which may have different configurations. In order to obtain our results we only modelled the risk of acquiring H1N1 based on airborne infection. During a flight, transmission due to direct contact is very limited because passengers remain seated. If the virus is also transmitted by large respiratory droplets (through direct contact) then the risks of infection that we have calculated will be underestimates and passengers in close proximity to the source case will be at greatest risk. We recommend that more detailed models of within-aircraft transmission of H1N1 are developed in order to investigate these issues. These models could be used to investigate the potential effectiveness of interventions such as the use of facial masks for symptomatic individuals.

We have demonstrated how methodology from the field of quantitative microbial risk assessment can be used to assess the importance of air travel on the global spread of H1N1. We used infectivity estimates from studies of seasonal strains of influenza to parameterize our model as several studies have now shown that H1N1 appears to have similar infectivity to seasonal strains [5,6]. We recommend, that in order to obtain further insights into the risks of acquiring influenza during air travel, the infectivity of all known strains of influenza (including H1N1) should be measured in terms of quanta/hour. In addition, we recommend estimating the infectivity of new strains of influenza as soon as they appear. As we have discussed in the introduction of this paper, there are already established methods for estimating infectivity. These methods measure infectivity in terms of infectious quanta by measuring virus RNA concentrations in exhaled respiratory droplets of infectious individuals [2].

Several mathematical models for predicting the potential spread and magnitude of a global pandemic of H1N1 have recently been published [16,17]. These large-scale models link H1N1 epidemics in different geographic regions by moving infectious individuals from place to place using air travel networks. They do not include the potential for transmission to occur during air travel. Based on our results, we suggest that hierarchical multi-scale network models are needed to more accurately predict the global dissemination of H1N1. Such models would expand on the current large-scale models based on air travel networks by linking them to small-scale models of H1N1 transmission within an aircraft. Such models would be hierarchical in nature as the small-scale models would be subcomponents of the large-scale models.

Our results have important implications for understanding and predicting the global dissemination of H1N1. They provide important insights into the potential utility of air travel restrictions on controlling influenza pandemics. We have shown that one infectious passenger could cause a significant number of H1N1 infections during long flights, particularly if they are travelling in Economy Class. These results imply that one individual travelling by plane, by infecting other travellers on the same flight, could cause multiple simultaneous outbreaks in different geographic locations rather than causing only one outbreak. Hence, quarantining passengers who travel in Economy Class on long-haul flights could potentially be an important control strategy in the winter of 2009/2010. Notably, our results show that quarantining passengers who travel First Class would be unlikely to be an effective control strategy.

## Competing interests

The authors declare that they have no competing interests.

## Authors' contributions

BGW, BJC and SB developed the concept and study design, analyzed and interpreted the data and drafted the manuscript. BGW and BJC conducted mathematical analyses. SB supervised the project.

## Pre-publication history

The pre-publication history for this paper can be accessed here:

http://www.biomedcentral.com/1741-7015/7/81/prepub

## References

[B1] Mangili A Gendreau MA Transmission of infectious diseases during commercial air travel Lancet 2005 365 989 996 10.1016/S0140-6736(05)71089-8 15767002 PMC7134995

[B2] Fabian P McDevitt JJ DeHaan WH Fung RO Cowling BJ Chan KH Leung GM Milton DK Influenza virus in human exhaled breath: an observational study PLoS ONE 2008 3 e2691 10.1371/journal.pone.0002691 18628983 PMC2442192

[B3] Lowen AC Mubareka S Tumpey T Garcia-Sastre A Palese P The guinea pig as a transmission model for human influenza viruses Proc Natl Acad Sci 2006 103 9988 9992 10.1073/pnas.0604157103 16785447 PMC1502566

[B4] Novel Swine-Origin Influenza A (H1N1) Investigation Team Dawood FS Jain S Finelli L Shaw M Lindstrom S Garten RJ Gubareva LV Bridges CB Uyeki TM Emergence of a novel swine-origin influenza A (H1N1) virus in humans N Engl J Med 2009 25 2605 2615 10.1056/NEJMoa0903810 19423869

[B5] Munster VJ de Wit E van den Brand JMA Herfst S Schrauwen EJA Bestebroer TM van de Vijver D Boucher CA Koopmans M Rimmelzwaan GF Pathogenesis and transmission of swine-origin 2009 A(H1N1) Influenza virus in ferrets Science 2009 325 481 483 19574348 10.1126/science.1177127 PMC4814155

[B6] Fraser C Donnelly CA Cauchemez S Hanage WP Van Kerkhove MD Hollingsworth TD Griffin J Baggaley RF Jenkins HE Lyons EJ Pandemic potential of a strain of influenza, A (H1N1): early findings Science 2009 19 1557 1561 10.1126/science.1176062 PMC3735127 19433588

[B7] Moser MR Bender TR Margolis HS Noble GR Kendal AP Ritter DG An outbreak of influenza aboard a commercial airliner Am J Epidemiol 1979 110 1 6 463858 10.1093/oxfordjournals.aje.a112781

[B8] Anderson RM May RM Infectious Diseases of Humans 1991 Oxford: Oxford Science Publications

[B9] Riley EC Murphy G Riley RL Airborne spread of measles in a suburban elementary school Am J Epidemiol 1978 107 421 432 665658 10.1093/oxfordjournals.aje.a112560

[B10] Wells WF On air-borne infection: II--Droplets and droplet nuclei Am J Hyg 1934 20 611 618

[B11] Liao CM Chang CF Liang HM A probabilistic transmission dynamic model to assess indoor airborne infection risks Risk Anal 2005 25 1097 1107 10.1111/j.1539-6924.2005.00663.x 16297217

[B12] Rudnick SN Milton DK Risk of indoor airborne infection transmission estimated from carbon dioxide concentration Indoor Air 2003 13 237 245 10.1034/j.1600-0668.2003.00189.x 12950586

[B13] Ryan PB Spengler JD Halfpenny PF Sequential box models for indoor air quality: Application to airliner cabin air quality Atmospheric Environment 1988 22 1031 1038 10.1016/0004-6981(88)90333-2

[B14] Boeing 747 Famiy: Technical Information http://www.boeing.com/commercial/747family/specs.html

[B15] Vander AJ Sherman JH Luciano DS Human Physiology 1994 New York: McGraw-Hill

[B16] Khan K Arino J Hu W Raposo P Sears J Calderon F Heidebrecht C Macdonald M Liauw J Chan A Gardam M Spread of a novel influenza A (H1N1) virus via global airline transportation N Engl J Med 2009 361 212 240 10.1056/NEJMc0904559 19564630

[B17] Balcan D Hu H Goncalves B Bajardi P Poletto C Ramasco JJ Paolotti D Perra N Tizzoni M Broeck W Van den Seasonal transmission potential and activity peaks of the new influenza A(H1N1): a Monte Carlo likelihood analysis based on human mobility BMC Med 2009 7 45 10.1186/1741-7015-7-45 19744314 PMC2755471

